# Trypsin-induced proteome alteration during cell subculture in mammalian cells

**DOI:** 10.1186/1423-0127-17-36

**Published:** 2010-05-11

**Authors:** Hsiang-Ling Huang, Hsiang-Wei Hsing, Tzu-Chia Lai, Yi-Wen Chen, Tian-Ren Lee, Hsin-Tsu Chan, Ping-Chiang Lyu, Chieh-Lin Wu, Ying-Chieh Lu, Szu-Ting Lin, Cheng-Wen Lin, Chih-Ho Lai, Hao-Teng Chang, Hsiu-Chuan Chou, Hong-Lin Chan

**Affiliations:** 1 Institute of Bioinformatics and Structural Biology & Department of Life Sciences, National Tsing Hua University, Hsinchu, Taiwan; 2 Department of Medical Laboratory Science and Biotechnology, China Medical University, Taichung, Taiwan; 3 Department of Microbiology, School of Medicine, China Medical University, Taichung, Taiwan; 4 Graduate Institute of Molecular Systems Biomedicine, China Medical University, Taichung, Taiwan; 5 Department of Applied Science, National Hsinchu University of Education, Hsinchu, Taiwan

## Abstract

**Background:**

It is essential to subculture the cells once cultured cells reach confluence. For this, trypsin is frequently applied to dissociate adhesive cells from the substratum. However, due to the proteolytic activity of trypsin, cell surface proteins are often cleaved, which leads to dysregulation of the cell functions.

**Methods:**

In this study, a triplicate 2D-DIGE strategy has been performed to monitor trypsin-induced proteome alterations. The differentially expressed spots were identified by MALDI-TOF MS and validated by immunoblotting.

**Results:**

36 proteins are found to be differentially expressed in cells treated with trypsin, and proteins that are known to regulate cell metabolism, growth regulation, mitochondrial electron transportation and cell adhesion are down-regulated and proteins that regulate cell apoptosis are up-regulated after trypsin treatment. Further study shows that bcl-2 is down-regulated, p53 and p21 are both up-regulated after trypsinization.

**Conclusions:**

In summary, this is the first report that uses the proteomic approach to thoroughly study trypsin-induced cell physiological changes and provides researchers in carrying out their experimental design.

## Background

Plasma membrane proteins are responsible for a wide variety of functions essential to maintaining normal physiological activities. For example, when EGF receptor families, a group of proteins located in the plasma membrane that act as growth receptors, transmit external signals into the cell interior, cell's physiological activities are often altered in response to external signals. In addition, adhesive proteins, such as the cadherin families [1] in the cell membrane, provide anchors to link cytoskeleton proteins with extracellular matrix to regulate cell migration and cell adhesion. The dysregulations of membrane proteins cause numerous diseases such as during tumorigenesis, malignant transformation of epithelial cells frequently attends with loss of E-cadherin expression and induction of expression of mesenchymal membrane proteins like N-cadherin [2,3]. Moreover, mutations of ErbB-2 receptors lead to the occurrence of gastric cancer [4] and hepatocellular cancer [5].

Two-dimensional gel electrophoresis (2-DE) has been widely used for profiling cellular proteins and some of the nonionic and zwitterionic detergents such as thiourea and CHAPS have been introduced to increase the solubility of the proteins. In addition, a significant improvement of gel-based analysis of protein quantifications and detections is the introduction of 2D-DIGE. 2D-DIGE is able to co-detect numerous samples in the same 2-DE to minimize gel-to-gel variation and compare the protein features across different gels by means of an internal fluorescent standard. This innovative technology relies on the pre-labeling of protein samples before electrophoresis with fluorescent dyes Cy2, Cy3 and Cy5 each exhibiting a distinct fluorescent wavelength to allow multiple experimental samples to include an internal standard. Thus, the samples can be simultaneously separated in one gel. The internal standard, which is a pool of an equal amount of the experimental protein samples, can facilitate the data accuracy in normalization and increase statistical confidence in relative quantitation across gels [6-10].

The primary step in adherent-cell-subculture is to detach cells from the substratum as the cells reach high confluence. Trypsin is often applied for this purpose. Cells are subsequently subdivided and reseeded into fresh cultures. However, the proteolytic activity of trypsin may harm cells by cleaving the cell surface growth factor receptors or membrane proteins. Hence, this study describes a 2D-DIGE strategy to perform cellular proteins labeling for the monitoring of trypsin-induced proteome alterations in mammalian cells.

## 2. Materials and Methods

### Chemicals and Reagents

Generic chemicals were purchased from Sigma-Aldrich (St. Louis, USA), while reagents for 2D-DIGE were purchased from GE Healthcare (Uppsala, Sweden). All primary antibodies were purchased from Abcam (Cambridge, UK) and secondary antibodies were purchased from GE Healthcare (Uppsala, Sweden). All chemicals and biochemicals used were of analytical grade. Fetal calf serum (FCS), antibiotics and trypsin were purchased from Invitrogen (all from Gibco-Invitrogen Corp., UK).

### Cell lines and cell cultures

The breast cancer cell line MCF-7 and cervical cancer cell line Hela were both purchased from American Type Culture Collection (ATCC), Manassas, VA. Both cell lines were maintained in Dulbecco's modified Eagle's medium (DMEM) supplemented with 10% (v/v) fetal calf serum (FCS), L-glutamine (2 mM), streptomycin (100 μg/mL), and penicillin (100 IU/mL) (all from Gibco-Invitrogen Corp., UK). Non-enzymatical cell dissociation solution was purchased from Sigma and 0.05% EDTA-Trypsin was purchased from Gibco-Invitrogen Corp. Cells were incubated in a humidified incubator at 37°C and 5% CO_2_.

### Cell trypsinization and CyDye labeling for 2D-DIGE analysis

The cellular protein labeling strategy was performed according to the protocol described previously with some modifications [9]. Once 90% of confluence is reached, MCF-7 and Hela cells were washed with Hank's balance salt solution (HBSS), detached with non-enzymatical cell dissociation solution and centrifuged for 5 min at 800 x g. The cell pellet was firstly washed with 1 ml ice cold HBSS pH8.3, and then resuspended in 200 μl of 2-DE lysis buffer containing 4% w/v CHAPS, 7 M urea, 2 M thiourea, 10 mM Tris-HCl, pH8.3 and 1 mM EDTA. Before performing 2D-DIGE, protein samples were labeled with N-hydroxy succinimidyl ester-derivatives of the cyanine dyes Cy2, Cy3 and Cy5. Briefly, 150 μg of protein sample was minimally labeled with 375 pmol of either Cy3 or Cy5 for comparison on the same 2-DE. To facilitate image matching and cross-gel statistical comparison, a pool of all samples was also prepared and labeled with Cy2 at a molar ratio of 2.5 pmol Cy2 per μg of protein as an internal standard for all gels. Thus, the triplicate samples and the internal standard could be run and quantify on multiple 2-DE. The labeling reactions were performed in the dark on ice for 30 min and then quenched with a 20-fold molar ratio excess of free L-lysine to dye for 10 min. The differentially Cy3- and Cy5-labeled samples were then mixed with the Cy2-labeled internal standard and reduced with dithiothreitol for 10 min. IPG buffer, pH3-10 nonlinear (2% (v/v), GE Healthcare) was added and the final volume was adjusted to 450 μl with 2D-lysis buffer for rehydration. The rehydration process was performed with immobilized non-linear pH gradient (IPG) strips (pH3-10, 24 cm) which were later rehydrated by CyDye-labeled samples in the dark at room temperature overnight (at least 12 hours). Isoelectric focusing was then performed using a Multiphor II apparatus (GE Healthcare) for a total of 62.5 kV-h at 20°C. Strips were equilibrated in 6 M urea, 30% (v/v) glycerol, 1% SDS (w/v), 100 mM Tris-HCl (pH8.8), 65 mM dithiothreitol for 15 min and then in the same buffer containing 240 mM iodoacetamide for another 15 min. The equilibrated IPG strips were transferred onto 26 x 20-cm 12.5% polyacrylamide gels casted between low fluorescent glass plates. The strips were overlaid with 0.5% (w/v) low melting point agarose in a running buffer containing bromophenol blue. The gels were run in an Ettan Twelve gel tank (GE Healthcare) at 4 Watt per gel at 10°C until the dye front had completely run off the bottom of the gels. Afterward, the fluorescence 2-DE was scanned directly between the low fluorescent glass plates using an Ettan DIGE Imager (GE Healthcare). This imager is a charge-coupled device-based instrument that enables scanning at different wavelengths for Cy2-, Cy3-, and Cy5-labeled samples. Gel analysis was performed using DeCyder 2-D Differential Analysis Software v7.0 (GE Healthcare) to co-detect, normalize and quantify the protein features in the images. Features detected from non-protein sources (e.g. dust particles and dirty backgrounds) were filtered out. Spots displaying a × 1.5 average-fold increase or decrease in abundance with a p-value < 0.05 were selected for protein identification.

### Protein staining

Colloidal coomassie blue G-250 staining was used to visualize Cy dye-labeled protein features in 2-DE according the protocol described in [11-13]. Briefly, bonded gels were fixed in 30% v/v ethanol, 2% v/v phosphoric acid overnight, washed three times (30 min each) with ddH_2_O and then incubated in 34% v/v methanol, 17% w/v ammonium sulphate, 3% v/v phosphoric acid for 1 hr., prior to adding 0.5 g/liter coomassie blue G-250. The gels were then left to stain for 5-7 days. No destaining step was required. The stained gels were then imaged on an ImageScanner III densitometer (GE Healthcare), which processed the gel images as .tif files.

### In-gel digestion

Excised post-stained gel pieces were washed three times with 50% acetonitrile, dried in a SpeedVac for 20 min., reduced with 10 mM dithiothreitol in 5 mM ammonium bicarbonate pH 8. 0 (AmBic) for 45 min at 50°C and then alkylated with 50 mM iodoacetamide in 5 mM AmBic for 1 hr. at room temperature in the dark. Gel pieces were then washed three times in 50% acetonitrile and vacuum-dried before reswelling with 50 ng of modified trypsin (Promega) in 5 mM AmBic. The pieces were then overlaid with 10 μl of 5 mM AmBic and trypsinized for 16 hr at 37°C. Supernatants were collected, peptides were further extracted twice with 5% trifluoroacetic acid in 50% acetonitrile and the supernatants pooled. Peptide extracts were vacuum-dried, resuspended in 5 μl ddH_2_O and stored at -20°C prior to MS analysis.

### Protein identification by MALDI-TOF MS

MALDI-TOF MS with generated peptide mass fingerprinting was used for protein identification. Briefly, 0.5 μl of tryptic digested protein sample was mixed with 0.5 μl of matrix solution containing α-cyano-4-hydroxycinammic acid at a concentration of 1 mg in 1 ml of 50% acetonitrile (v/v)/0.1% trifluoroacetic acid (v/v), spotted onto a anchorchip target plate (Bruker Daltonics) and dried. The peptide mass fingerprints were acquired using an Autoflex III mass spectrometer (Bruker Daltonics) in reflector mode. The spectrometer was calibrated with a peptide calibration standard (Bruker Daltonics) and internal calibration was performed using trypsin autolysis peaks at *m/z *842.51 and *m/z *2211.10. Peaks in the mass range *m/z *800-3000 were used to generate a peptide mass fingerprint that was searched against the updated Swiss-Prot/TrEMBL database (v56.5) with 402482 entries on December 19, 2008 using Mascot software v2.2.04 (Matrix Science, London, UK). The parameters used for the search were: *Homo sapiens*; tryptic digest with a maximum of 1 missed cleavage; carbamidomethylation of cysteine, partial protein N-terminal acetylation, partial methionine oxidation and partial modification of glutamine to pyroglutamate and a mass tolerance of 50 ppm. Identification was accepted based on significant MASCOT Mowse scores (*p *< 0.05).

### Immunoblotting

Immunoblotting was used to validate the differential expression of mass spectrometry identified proteins. Membrane fraction extracts were briefly lysed with 2-DE lysis buffer prior to protein quantification with Coomassie Protein Assay Reagent (BioRad). 30 μg of protein samples were diluted in Laemmli sample buffer (final concentrations: 50 mM Tris pH 6.8, 10% (v/v) glycerol, 2% SDS (w/v), 0.01% (w/v) bromophenol blue) and separated by 1D-SDS-PAGE according to standard procedures. After electroblotting of separated proteins onto 0.45 μm Immobilon P membranes (Millipore), the membranes were blocked with 5% w/v skimmed milk in TBST (50 mM Tris pH 8.0, 150 mM NaCl and 0.1% Tween-20 (v/v)) for 1 hr. Membranes were then incubated in primary antibody solution in TBS-T containing 0.02% (w/v) sodium azide for 2 hrs. Membranes were washed in TBS-T (3 x 10 min) and then probed with the appropriate horseradish peroxidase-coupled secondary antibody (GE Healthcare). After further washes in TBS-T, immunoprobed proteins were visualized using an enhanced chemiluminescence method (Visual Protein Co.).

## Results

### 2D-DIGE analysis of the trypsin-induced differentially expressed proteins

**Figure 1:**
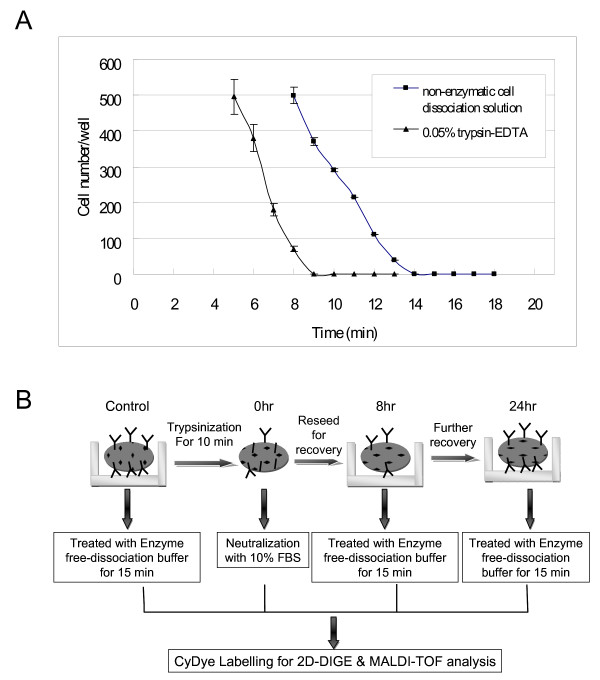
**Cell dissociation workflow with and without trypsin digestion**. (A) The dissociation time for attached MCF-7 cells were determined where 90% confluent cells in 96-well cell culture plates were gently washed with HBSS twice followed by treated with 100 μl of non-enzymatic cell dissociation solution or 0.05% trypsin-EDTA. After indicated treatment times, cells were gently washed with HBSS and the number of adherent cells counted. The mean cell number of 4 independent assays is shown +/- SD. (B) Overview of cell dissociation workflow with and without trypsin digestion of adhesive MCF-7 cells.

**Figure 2:**
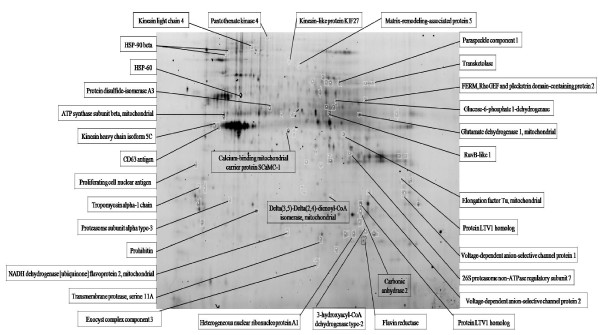
**2D-DIGE analysis of the trypsin-induced differentially expressed proteins in MCF-7 cells**. Protein samples purified from total cell lysates were labeled with Cy dyes and separated using 24-cm, pH 3-10 nonlinear IPG strip. The differentially expressed protein features are annotated with protein names. The detail information for these identified proteins is listed in Additional file 1.

To identify the altered abundance of proteins and relate them to trypsinization, MCF-7 cells were washed with HBSS followed by dissociating cells from substratum with non-enzymatic cell dissociation solution for 15 min or 0.05% trypsin-EDTA for 10 min after cells reach approximately 90% confluence. The dissociation time for attached MCF-7 cells were optimized by examined the number of adherent cells after treatment of non-enzymatic cell dissociation solution or 0.05% trypsin-EDTA (Figure 1A). Trypsin-digested MCF-7 cells were then either directly neutralized with 10% FCS followed by performed cell lysis or reseeded onto cell culture plates to recover for 8 hr and 24 hr before being dissociated with non-enzymatic cell dissociation solution for cell lysis. Subsequently, the lysed cells from each condition were minimally labeled with Cy3 or Cy5 dye and distributed to each gel. A pool of all samples was also prepared for labeling with Cy2 as an internal standard to run on all gels to facilitate image matching across gels. Thus, the triplicate samples resolved in different gels can be quantitatively analyzed by means of the internal standard on multiple 2-DE. The dissociation and protein labeling procedures are described in Figure 1B and in the Materials & Methods section. After resolving protein samples with 2D-DIGE technique, the DeCyder image analysis software indicated that more than 60 protein features were showing greater than 1.5-fold change in expression level. MALDI-TOF MS identification revealed that 36 proteins were differentially expressed during trypsinization (Figure 2 and Additional file 1). Most of these identified proteins are located in the cytoplasm (39%), mitochondria (25%) and the plasma membrane (22%), and these proteins are found to be involved in growth regulation (23%), metabolism (11%) and vascular transportation (11%).

### Validation of identified proteins by immunoblotting

**Figure 3:**
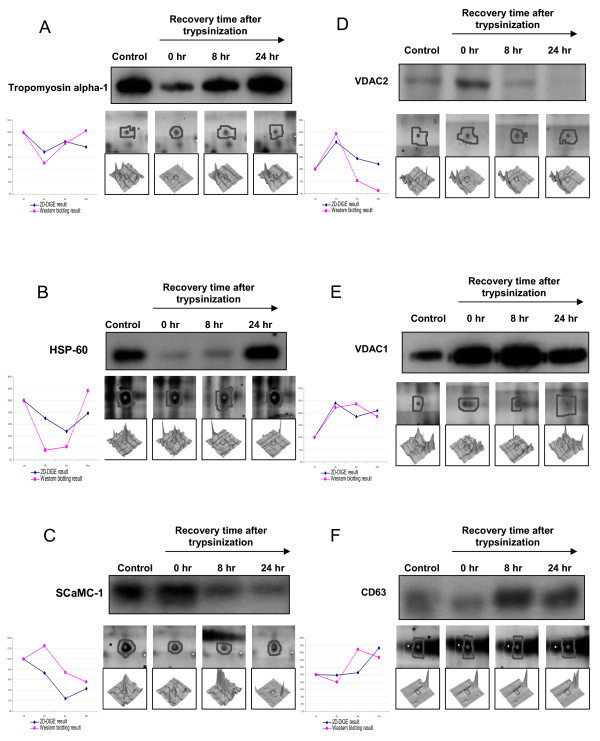
**Representative immunoblotting analysis for selected differentially expressed proteins during trypsinization**. The levels of identified proteins (A) tropomyosin alpha-1, (B) HSP-60, (C) SCaMC-1, (D) VDAC2, (E) VDAC1, and (F) CD63 in MCF-7 cells were analyzed by immunoblotting (left top panels), protein expression map from 2D-DIGE (left middle panels), three-dimensional spot images (left bottom panels) and relative quantification of western blotting and 2D-DIGE data for each target protein (right panels).

Immunoblot analysis was carried out to confirm the expression levels of the following differentially expressed proteins (tropomyosin alpha-1, HSP-60, SCaMC-1, VDAC2, VDAC1 and CD63) observed in 2D-DIGE (Figure 3). The immunoblotting validation indicated that tropomyosin alpha-1 and HSP-60 decreased at 0 hr of trypsinization, but were restored after 24 hr. This result was consistent with the proteomic analysis. Protein expression levels of SCaMC-1 and CD63 were down-regulated and up-regulated, respectively at hour 8 after trypsinization, and their expression levels did not return to the control levels even after 24 hr. In another observation, both VDAC1 and VDAC2 were up-regulated at the time of trypsinization and recovered to basal level at around 24 hr and 8 hr, respectively, which were both consistent with the 2D-DIGE results.

### Detection of trypsin-induced differential protein expression in cervical cancer cells

**Figure 4:**
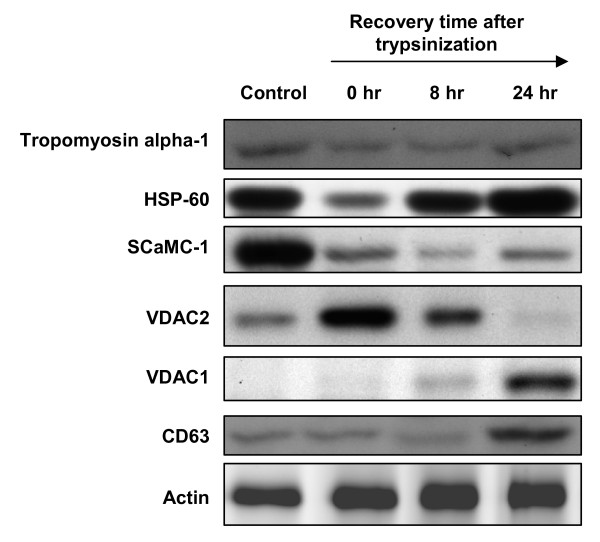
**Expression level analysis of tropomyosin alpha-1, HSP-60, SCaMC-1, VDAC1, VDAC2 and CD63 during trypsinization in Hela cells**.

It is interesting and important to know whether trypsine-induced protein alterations in MCF-7 cells are reproducible in other cell types. Accordingly, cervical adenocarcinoma Hela cells were used to examine protein expression changes by trypsinization (Figure 4). Immunoblotting analysis indicated that tropomyosin alpha-1 was slightly decreased at 0 hr and 8 hr of trypsinization and was slightly restored after 24 hr. In contrast, HSP-60 significantly decreased at 0 hr of trypsinization and was completely restored after 24 hr. This result is consistent with the proteomic analysis found in MCF-7 cells. Protein expression level of SCaMC-1 was down-regulated soon after trypsinization, and the expression level did not return to the control level even after 24 hr. This implies SCaMC-1 may need a longer period of time for recovery and the result is highly consistent with the proteomic analysis found in MCF-7 cells. Furthermore, VDAC2 and CD63 were up-regulated at the time soon after trypsinization and at 24 hr, respectively; which is also consistent with the previous results found in MCF-7 cells. In contrast to an instantly up-regulated VDAC1 level was shown in MCF-7 cells, a significantly enhanced VDAC1 level in Hela cells starting from 8 hr. In summary, trypsinization-induced protein alterations in MCF-7 cells are highly correlated to Hela cells except for Tropomyosin alpha-1 and VDAC1.

### Functional expression profiles of the identified differentially expressed proteins

**Figure 5:**
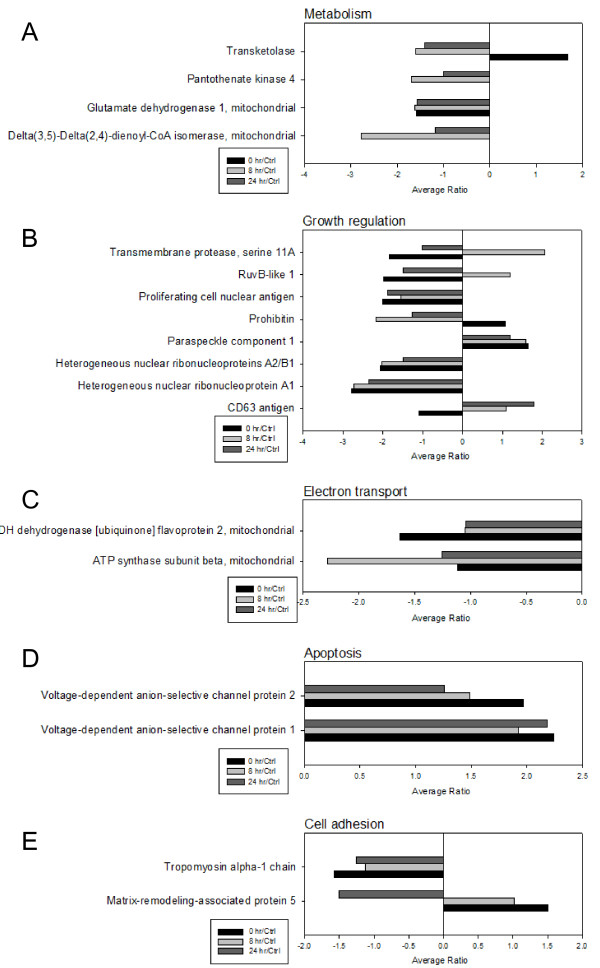
**Expression profiles for proteins potentially contributing to (A) metabolism (B) growth regulation (C) electron transportation (D) apoptosis (E) cell adhesion in comparing MCF-7 cells treated with 0.05% trypsin for 10 min followed by recovery for 0 hr, 8 hr and 24 hr or left untreated**. The horizontal bars represent fold change in protein expression and the vertical axis indicates the identified proteins. Additional details for each protein can be found in Additional file 1.

**Figure 6:**
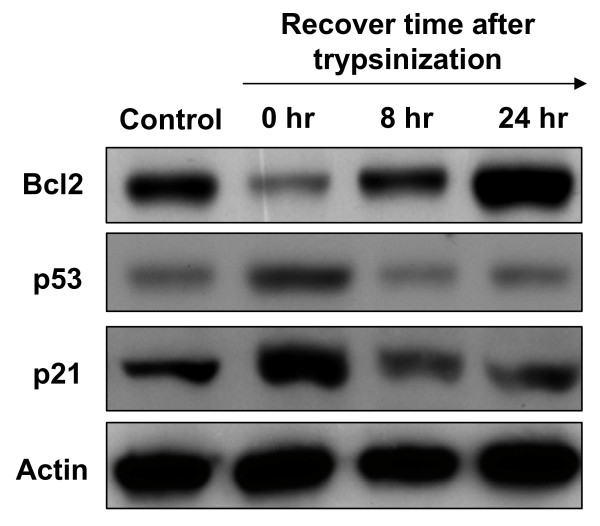
**Comparison of trypsin-induced cell signaling in cell survival, apoptosis and cell cycle regulation**. Activation of survival, apoptosis and cell cycle inhibition signalling pathways were analyzed by immunoblotting with anti-Bcl2, anti-p53 and anti-p21 antibodies, respectively.

With the basis of a Swiss-Prot search and KEGG pathway analysis, numerous potential biological functions of the identified proteins in MCF-7 cells which were treated with trypsin and then recovered for 0 hr, 8 hr or 24 hr or left untreated were determined. Proteins known to regulate cell metabolism, growth regulation, mitochondrial electron transportion and cell adhesion were found to be down-regulated in trypsinized MCF-7 cells even after a 24-hr recovery in fresh medium (Figure 5A, B, C and 5E). In contrast, proteins known to regulate cell apoptosis were shown to be up-regulated in trypsinized MCF-7 cells after a 24-hr recovery (Figure 5D). These proteomic results indicated that trypsinization might decrease growth- and metabolism-related protein expression levels and slightly increase apoptosis-related proteins. To further examine this observation, trypsin-induced cell signals in cell survival, apoptosis and cell cycle regulation were verified by immunoblotting. The result showed the cell survival marker, Bcl-2, was down-regulated; on the other hand, the cell apoptotic marker, p53, and cell cycle inhibitor, p21, were both up-regulated during trypsinization (Figure 6).

## Discussion

Differential protein expression in adhesive cell subculture with trypsin has not been discussed thoroughly in previous studies. However, due to the proteolytic activity of trypsin, membrane proteins of the cell might be damaged which results in cellular dysfunctions. Hence, a CyDye labeling 2D-DIGE technique, along with MALDI-TOF MS identification, was performed in this study, and 36 proteins revealed a significant expression change due to trypsinization. Moreover, the proteomic results demonstrated that trypsinization down-regulated growth- and metabolism-related protein expressions and up-regulated apoptosis-related protein expressions. These findings implied that trypsin used for cell subculture had a remarkable adverse effect on cell physiology. Notably, some of these trypsin-induced differentially expressed proteins were reversible while a portion of these proteins remains dysregulated even after a 24-hr recovery in fresh medium.

Proteomic analysis of the trypsin-induced differentially expressed proteins in MCF-7 indicated that numerous differentially expressed proteins are involved in the chaperon functions (HSP-60, HSP-90 beta, Protein disulfide-isomerase A3) implying trypsinization might induce a stress response on culture cells. These chaperon proteins have been reported to be cell surface located [14-16] and cell surface located chaperons also play crucial roles in mediating integrin activations in breast cancer cells [17]. In addition, one of the most important findings in this study is that trypsinization decreases the growth- and metabolism-related protein expression levels and increases the apoptosis-related protein expression levels. This observation is not only confirmed by the immunoblotting result (Figure 6), but also verified by MALDI-TOF, which indicate that trypsinization decreases the expression levels of proteins involved in DNA replication (Proliferating cell nuclear antigen and RuvB-like 1), RNA splicing (Heterogeneous nuclear ribonucleoprotein A1 and Heterogeneous nuclear ribonucleoproteins A2/B1) and mitochondria metabolism (3-hydroxyacyl-CoA dehydrogenase type-2, ATP synthase subunit beta, Calcium-binding mitochondrial carrier protein SCaMC-1, Delta(3,5)-Delta(2,4)-dienoyl-CoA isomerase, Glutamate dehydrogenase 1 and NADH dehydrogenase). In contrast, trypsinization promotes the overexpression of voltage-dependent anion-selective channel protein 1 and voltage-dependent anion-selective channel protein 2. These proteins play essential roles in the increase of mitochondrial membrane permeability and lead to cell apoptosis [18] (Figure 5 and Additional file 1). Importantly, it is essential to know whether the trypsinization-induced protein alterations in MCF-7 cells are commonly recognized in other cell types. Therefore, the other cell line, Hela cell was used for further investigation and the results showed that trypsinization-induced protein alterations in MCF-7 cells are mostly comparable in Hela cells.

In conclusion, 2D-DIGE based proteomics analysis serves as a useful tool to monitor trypsin-induced cell proteome alterations in this study. Trypsinization has shown to down-regulate growth- and metabolism-related protein expression and up-regulate apoptosis-related protein expression. This study helps researchers who work in the cell signaling and cell biology fields to carefully examine the impact of trypsin in carrying out their experimental design.

## Abbreviations

1-DE: one-dimensional gel electrophoresis; 2-DE: two-dimensional gel electrophoresis; Ab: antibody; AmBic: ammonium bicarbonate; CCB: colloidal coomassie blue; CHAPS: 3- [(3-cholamidopropyl)-dimethylammonio]-1-propanesulfonate); DIGE: differential gel electrophoresis; DTT: dithiothreitol; FCS: fetal calf serum; IAM: iodoacetamide; MALDI-TOF MS: matrix assisted laser desorption ionization-time of flight mass spectrometry; TFA: trifluoroacetic acid.

## Competing interests

The authors declare that they have no competing interests.

## Authors' contributions

HLC and HCC designed the experiments and the draft manuscript writing. HLH, HWH, TCL, YWC, CLW, YCL, STL performed the cell culture, 2D-gel electrophoresis, image analysis and immunoblotting. HLC and HCC supervised the experiments and the data analysis. TRL, PCL, CWL, CHL, HTC contributed to the data interpretation and the data discussion. HLC, HTC and HCC finalized the manuscript. All authors have read and approved the final manuscript.

## Supplementary Material

Additional file 1 **Alphabetical list of trypsin digestion-induced differentially expressed proteins identified by MALDI-TOF peptide mass fingerprinting after 2D-DIGE analysis**. ^a^not analyzed; ^b^subcellular location and functional classification of identified proteins were referred to the Uniprot website http://www.uniprot.org/. Click here for file

## References

[B1] Peralta SA Knudsen KA Tecson-Miguel A McBrearty FX Han AC Salazar H Expression of E-cadherin and N-cadherin in surface epithelial-stromal tumors of the ovary distinguishes mucinous from serous and endometrioid tumors Hum Pathol 1997 28 734 739 10.1016/S0046-8177(97)90184-2 9191009

[B2] Rosivatz E Becker I Bamba M Schott C Diebold J Mayr D Hofler H Becker KF Neoexpression of N-cadherin in E-cadherin positive colon cancers Int J Cancer 2004 111 711 719 10.1002/ijc.20317 15252840

[B3] Kuphal S Bosserhoff AK Influence of the cytoplasmic domain of E-cadherin on endogenous N-cadherin expression in malignant melanoma Oncogene 2006 25 248 259 10.1038/sj.onc.1209508 16132038

[B4] Lee JW Soung YH Kim SY Park WS Nam SW Kim SH Lee JY Yoo NJ Lee SH ERBB2 kinase domain mutation in a gastric cancer metastasis APMIS 2005 113 683 687 16309427 10.1111/j.1600-0463.2005.apm_284.x

[B5] Bekaii-Saab T Williams N Plass C Calero MV Eng C A novel mutation in the tyrosine kinase domain of ERBB2 in hepatocellular carcinoma BMC Cancer 2006 6 278 10.1186/1471-2407-6-278 17150109 PMC1712353

[B6] Timms JF Cramer R Difference gel electrophoresis Proteomics 2008 8 4886 4897 10.1002/pmic.200800298 19003860

[B7] Westermeier R Scheibe B Difference gel electrophoresis based on lys/cys tagging Methods Mol Biol 2008 424 73 85 full_text 18369854 10.1007/978-1-60327-064-9_7

[B8] Marouga R David S Hawkins E The development of the DIGE system: 2D fluorescence difference gel analysis technology Anal Bioanal Chem 2005 382 669 678 10.1007/s00216-005-3126-3 15900442

[B9] Lai TC Chou HC Chen YW Lee TR Chan HT Shen HH Lee WT Lin ST Lu YC Wu CL Chan HL Secretomic and Proteomic Analysis of Potential Breast Cancer Markers by Two-Dimensional Differential Gel Electrophoresis J Proteome Res 2010 9 1302 1322 10.1021/pr900825t 20052998

[B10] Chou HC Chen YW Lee TR Wu FS Chan HT Lyu PC Timms JF Chan HL Proteomics study of oxidative stress and Src kinase inhibition in H9C2 cardiomyocytes: A cell model of heart ischemia reperfusion injury and treatment Free Radic Biol Med 2010 in press 20385227 10.1016/j.freeradbiomed.2010.04.001

[B11] Chan HL Gaffney PR Waterfield MD Anderle H Peter MH Schwarz HP Turecek PL Timms JF Proteomic analysis of UVC irradiation-induced damage of plasma proteins: Serum amyloid P component as a major target of photolysis FEBS Lett 2006 580 3229 3236 10.1016/j.febslet.2006.05.002 16697377

[B12] Chan HL Gharbi S Gaffney PR Cramer R Waterfield MD Timms JF Proteomic analysis of redox- and ErbB2-dependent changes in mammary luminal epithelial cells using cysteine- and lysine-labelling two-dimensional difference gel electrophoresis Proteomics 2005 5 2908 2926 10.1002/pmic.200401300 15954156

[B13] Chan HL Chou HC Duran M Gruenewald J Waterfield MD Ridley A Timms JF Major role of EGFR and SRC kinases in promoting oxidative stress-dependent loss of adhesion and apoptosis in epithelial cells J Biol Chem 2009 285 4307 4318 10.1074/jbc.M109.047027 19996095 PMC2836035

[B14] Shin BK Wang H Yim AM Le Naour F Brichory F Jang JH Zhao R Puravs E Tra J Michael CW Misek DE Hanash SM Global profiling of the cell surface proteome of cancer cells uncovers an abundance of proteins with chaperone function J Biol Chem 2003 278 7607 7616 10.1074/jbc.M210455200 12493773

[B15] Jang JH Hanash S Profiling of the cell surface proteome Proteomics 2003 3 1947 1954 10.1002/pmic.200300563 14625857

[B16] Mayrhofer C Krieger S Allmaier G Kerjaschki D DIGE compatible labelling of surface proteins on vital cells in vitro and in vivo Proteomics 2006 6 579 585 10.1002/pmic.200500104 16372259

[B17] Barazi HO Zhou L Templeton NS Krutzsch HC Roberts DD Identification of heat shock protein 60 as a molecular mediator of alpha 3 beta 1 integrin activation Cancer Res 2002 62 1541 1548 11888933

[B18] Tsujimoto Y Shimizu S The voltage-dependent anion channel: an essential player in apoptosis Biochimie 2002 84 187 193 10.1016/S0300-9084(02)01370-6 12022949

