# Editorial expression of concern: Trypsin-induced proteome alteration during cell subculture in mammalian cells

**DOI:** 10.1186/s12929-026-01285-4

**Published:** 2026-08-24

**Authors:** Hsiang-Ling Huang, Hsiang-Wei Hsing, Tzu-Chia Lai, Yi-Wen Chen, Tian-Ren Lee, Hsin-Tsu Chan, Ping-Chiang Lyu, Chieh-Lin Wu, Ying-Chieh Lu, Szu-Ting Lin, Cheng-Wen Lin, Chih-Ho Lai, Hao-Teng Chang, Hsiu-Chuan Chou, Hong-Lin Chan

**Affiliations:** 1 Institute of Bioinformatics and Structural Biology & Department of Life Sciences, National Tsing Hua University, Hsinchu, Taiwan; 2 Department of Medical Laboratory Science and Biotechnology, China Medical University, Taichung, Taiwan; 3 Department of Microbiology, School of Medicine, China Medical University, Taichung, Taiwan; 4 Graduate Institute of Molecular Systems Biomedicine, China Medical University, Taichung, Taiwan; 5 Department of Applied Science, National Hsinchu University of Education, Hsinchu, Taiwan

**Editorial expression of concern: Journal of Biomedical Science 2010, 17:36** 10.1186/1423-0127-17-36

The Editors would like to alert the readers that concerns have been raised regarding the Figure 4. Specifically, Figure 4 VDAC2 appears to be very similar with Figure 1A pp38 in a previous publication [1]. The authors are not able to provide original data so the images could not be verified. Readers are advised to interpret these results with caution.

Authors Hsiang-Ling Huang and Hong-Lin Chan agree to this Editorial Expression of Concern. The rest of the authors have not responded to correspondence regarding this Editorial Expression of Concern.
